# Soluble MICB in Plasma and Urine Explains Population Expansions of NKG2D^+^CD4 T Cells Inpatients with Juvenile-Onset Systemic Lupus Erythematosus

**DOI:** 10.4236/oji.2017.71001

**Published:** 2017-03-29

**Authors:** Satoru Hamada, Andrea Caballero-Benitez, Kate L. Duran, Anne M. Stevens, Thomas Spies, Veronika Groh

**Affiliations:** 1 Clinical Research Division, Fred Hutch, Seattle, WA, USA; 2 Department of Pediatrics, Ryukyus University, Okinawa Prefecture, Nishihara, Japan; 3 Division of Rheumatology, Department of Pediatrics, University of Washington Medicine, Seattle, WA, USA; 4 Center for Immunity and Immuno Therapies, Seattle Children’s Research Institute, Seattle, WA, USA

**Keywords:** NKG2D Ligands, NKG2D^+^ CD4 T Cells, Juvenile-Onset Systemic Lupus Erythematosus, B Cells, Monocytes

## Abstract

Abnormal NKG2D ligand expression has been implicated in the initiation and maintenance of various auto-inflammatory disorders including systemic lupus erythematosus (SLE). This study’s goal was to identify the cellular contexts providing NKG2D ligands for stimulation of the immunosuppressive NKG2D^+^CD4 T cell subset that has been implicated in modulating juvenile-onset SLE disease activity. Although previous observations with NKG2D^+^CD4 T cells in healthy individuals pointed towards peripheral B cell and myeloid cell compartments as possible sites of enhanced NKG2DL presence, we found no evidence for a disease-associated increase of NKG2DL-positivity among juvenile-onset SLE B cells and monocytes. However, juvenile-onset SLE patient plasma and matched urine samples were positive by ELISA for the soluble form of the NKG2D ligands MICA and MICB, suggesting that kidney and/or peripheral blood may constitute the NKG2DL positive microenvironments driving NKG2D^+^CD4 T cell population expansions in this disease.

## 1. Introduction

The NKG2D lymphocyte receptor and its cell stress-induced ligands have been implicated in the initiation and amplification of several autoimmune and inflammatory disorders [1]. In humans, NKG2D is expressed by most natural killer (NK) cells and CD8 T cells, and some CD4 T cells [2] [3]. NKG2D signal via association with the tyrosinemotive-containing DAP10 adapter and, upon ligand binding, stimulates effector functions or proliferation and survival [2] [4] [5]. NKG2D ligands (NKG2DL) include the MHC class I-related chains A and B (MICA, MICB) and six members of the UL-16 binding protein family (ULBP1-6) [6]. Central to the immunobiology of these ligands and, by inference of NKG2D, is the fact that functionally relevant amounts are absent from the surface of most normal cells but induced by mechanisms associated with cellular stress responses and thus frequently present on transformed or infected cells, and at sites of tissue inflammation [7].

Aberrant NKG2DL presence occurs in autoimmune and inflammatory diseases such as rheumatoid arthritis [8], alopecia areata [9], and celiac and Crohn’s disease [10] [11]. In all these conditions, ligand engagement of NKG2D on tissue-resident effector lymphocytes promotes cell damage and inflammation. Abnormalities in NKG2D-mediated immune responses have also been implicated in the pathophysiology of systemic lupus erythematosus (SLE), although with immune-modulatory rather than tissue damage-promoting consequences [3]. Juvenile-onset SLE (jSLE) disease activity negatively correlates with population expansions of a normally rare CD4 T cell subset that expresses NKG2D, is auto-reactive, and has immunosuppressive functions [3]. Large numbers of such NKG2D^+^CD4 T cells also occur within cancer tissues where autoantigens and NKG2DL on tumor cells enable NKG2D-costimulated T cell proliferation [12]. With lupus, the ligand-positive cellular contexts providing for NKG2D engagement remain largely unknown. This study aims at addressing this knowledge gap.

## 2. Materials and Methods

### 2.1. Subjects and Blood Samples

19 patients diagnosed before age 18 who fulfilled the American College of Rheumatology criteria for the classification of SLE [13] and age-matched healthy volunteers were included into the study. Patients and healthy volunteers were recruited locally by word of mouth. Demographic characteristics, disease activity and renal disease status, and immunomodulatory medications of all patients are listed in Table 1. Control group demographic data other than age (range 11.8 – 18) was not available. Peripheral blood, and clinical and laboratory data were collected for each patient and disease activity determined according to the modified SLEDAI Index 2000 [14]. Disease activity scores of five or greater were considered representing active, scores of less than five inactive disease [15] [16]. Sample procurement from jSLE033-122 was between 042006 and 062012; from CIIT-1001-20 between 082015 and 012016. Serial samples obtained at times of high or low disease activity were available from three patients. PBMC were isolated as described [3]. Paired urine and plasma samples were collected from an additional 5 jSLE patients (Table 1). All activities were carried out in accordance with the Declaration of Helsinki and approved by local Institutional Review Boards. All subjects provided informed written consent.

### 2.2. Polychromatic Flow Cytometry

PBMC staining and analysis were as described [3]. Unless otherwise specified, mAbs were from BD Pharmingen and included anti-CD3-Alexa Fluor 700 (UC-HT1), anti-CD4-APC (RPA-T4), anti-CD8-PacificBlue (RPA-T8), anti-CD11c-FITC (BU15, Thermo Scientific), anti-CD14-APC (RMO52, Beckman Coulter), anti-CD16-PacificBlue (3G8), anti-CD19-APC (HIB19) and anti-CD20-FITC (L27), anti-CD56-APC (N901, Beckman Coulter), and anti-NKG2D-PE (1D11; [2]). Anti-NKG2DL mAb were anti-MICA/B-PE (6D4), anti-ULBP1-PE (170818, R & D Systems), anti-ULBP2/5/6-PE (165903; R & D Systems), anti-ULBP3-PE (166510; R & D Systems), and anti-ULBP4-PE (1H1; [12]). In some cases, viable cell numbers were limiting resulting in partial data sets.

### 2.3. Monocyte Stimulation

Monocytes, enriched from either patient or healthy donor PBMC using classic plastic adherence, were incubated with RMPI-1640/10% FBS supplemented with LPS (10 μg/ml; Sigma) or GM-CFS (20 ng/ml; R & D Systems), or with medium alone, for 24 hours and examined for NKG2DL expression by flow cytometry.

### 2.4. ELISA for Soluble NKG2D Ligand Detection

Soluble MICA (sMICA), soluble MICB (sMICB), and soluble ULBP1 (sULBP1) in jSLE patient plasma were determined using ELISA kits (Human MICA and Human MICB; Abcam; Human ULBP-1, R & D Systems) according to manufacturers’ instructions.

### 2.5. Statistical Analysis

Populations were compared using the two-sample *t*-test and significance assigned where *p* < 0.05. Contribution of more than one sample by some subjects was accounted for using multivariate logistic generalized estimating equations (GEE). The correlation between NKG2D^+^CD4 T cell frequencies and soluble NKG2DL was estimated from linear regression.

## 3. Results

### 3.1. Juvenile-Onset SLE B Cells Express Normal NKG2D Ligand Profiles

Our previous observation of lupus-associated NKG2D^+^CD4 T cell population expansions was made in patients with the juvenile-onset form of this disease [3]. For consistency, we maintained this disease focus enrolling 19 jSLE patients and 20 healthy age-matched control (HC) donors. Flow cytometry of PBMC samples (altogether 22, since each two serial samples were available from three patients; Table 1) confirmed increased frequencies of NKG2D^+^CD4 T cells among the jSLE cohort compared to HC (10.82% ± 8.72% versus 3.64% ± 2.34; *p* ≤ 0.00001) with larger proportions of these cells in inactive compared to active disease (15.99% ± 10.48% versus 6.12% ± 3.47; *p* = 0.0007).

**Figure 1:**
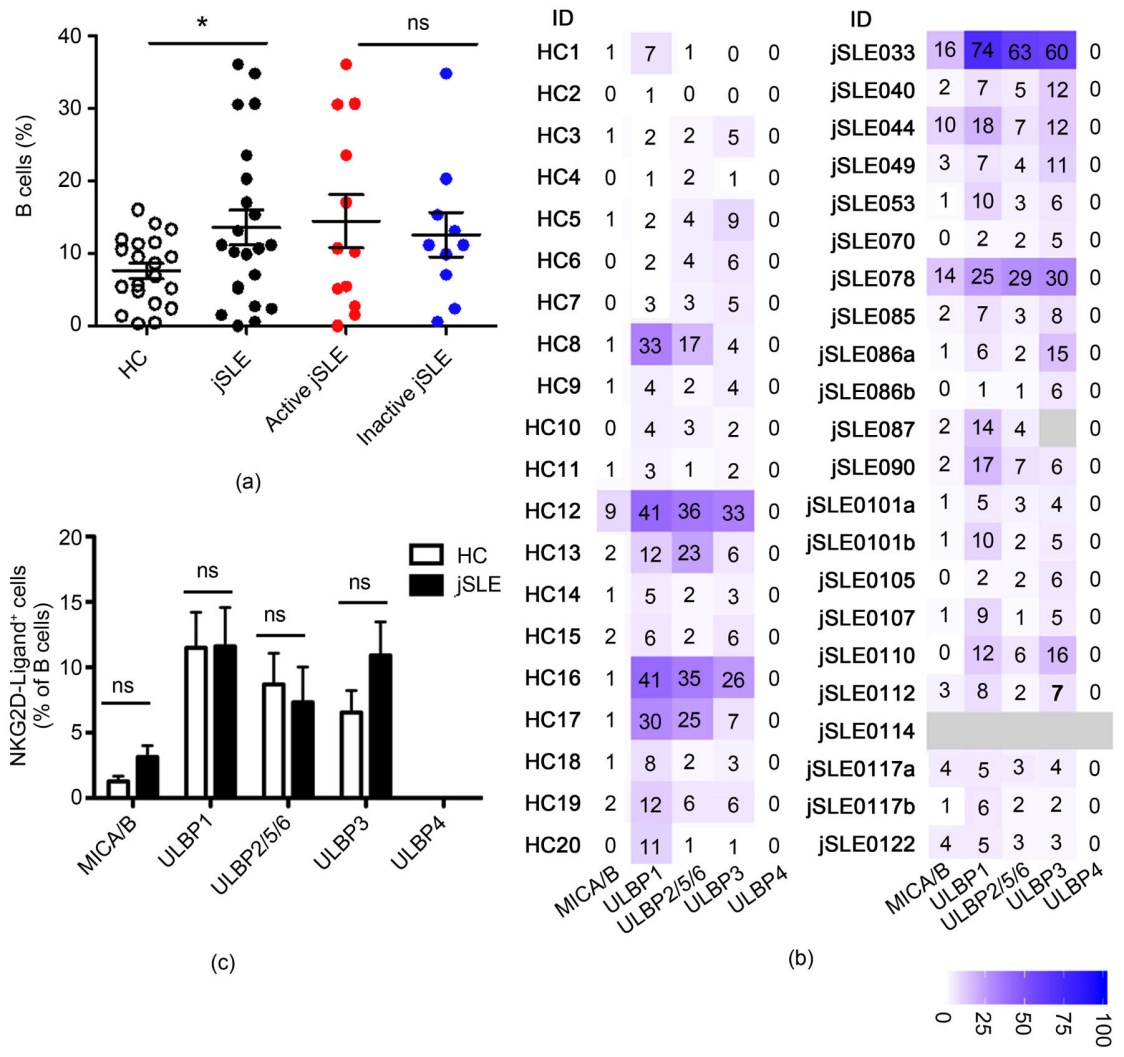
Frequencies and NKG2DL phenotypes of peripheral blood B cells in juvenile-onset SLE patients and healthy controls (HC). (a) Comparisons of proportions (% of total lymphocytes) of B cells in HC to those in jSLE patients, and between active and inactive disease. Horizontal lines and error bars show median and interquartile range. (b) Heat map display of proportions (numbers in individual squares) of B cells expressing the indicated NKG2D ligands in each patient and control sample. Light grey indicates no data; bar displays color grading; (ID) identification. (c) Graphic display of proportions of B cells expressing the indicated ligands. (a)–(c) *p < 0.05; (ns) not significant.

In healthy individuals, peripheral blood B cells provide the autoantigens and NKG2DL necessary for NKG2D^+^CD4 T cell proliferation [3]. Screening for NKG2DL expression in jSLE thus first focused on the peripheral B cell compartment. Lupus patients, adult and juvenile-onset alike, are lymphopenic and, compared to healthy individuals, have reduced absolute B cell counts [17]. Accommodating these contractions and the typically small volumes of pediatric blood draws we started out with an exploratory flow cytometry analysis that defined B cells based only on CD19 and CD20 coexpression without consideration of additional functionally relevant markers. NKG2DL were tested as a third parameter using mAbs to MICA/B, ULBP1, ULBP2/5/6, ULBP3, and ULBP4 (Supplementary Figure S1 displays examples of gating strategy and primary data). CD19^+^CD20^+^ B cells were present in all but one (jSLE114) of the 22 jSLEPBMC samples with absolute counts lower than those described for healthy children (219.8 ± 282.4 cells/μl; [17] [18]). Relative proportions of CD19^+^CD20^+^ cells were larger than those of the control donors (mean 13.6% ± 11.3 versus 7.6% ± 4.7; *p* = 0.0187) but independent of disease activity (Figure 1(a)).

NKG2DL expression profiles of jSLE B cells displayed pronounced individual variability and, overall, were similar to those recorded with HC (Figure 1(b) and Figure 1(c); [19] [20]). In both cohorts, ULBP ligands (except ULBP4) were more prevalent than MICA/B. B cells expressing at least one, and most of the time two or more NKG2DL were present in all samples, albeit in ~50% of jSLE and HC samples their frequencies were low (<10% of total CD19^+^CD20^+^ cells). 10% or more NKG2DL-positive B cells were detected in 11 jSLE patients and 7 HC. In most samples, two or more NKG2DL were expressed at similar frequencies suggesting coordinate expression of more than one ligand by a given cell (Figure 1(b)). Indeed, co-staining for ULBP1 and ULBP2/5/6 confirmed co-expression of these ligands on CD19^+^CD20^+^ B cells in three additional healthy donor PBMC samples (data not shown). There were no correlations between frequencies of NKG2DL-positive B cells and frequencies of NKG2D^+^CD4 T cells (data not shown). Thus although, as with normal B cells, jSLE B cells, if NKG2DL-positive, may well contribute to NKG2D^+^CD4 T cell proliferation, it seems unlikely that the jSLEB cell compartment alone provides the NKG2DL abundance presumed necessary for the extensive proliferative expansions of NKG2D^+^CD4 T cell populations that occur in jSLE [3].

### 3.2. Reduced Frequencies of NKG2D Ligand-Positive Cells among Juvenile-Onset SLE Monocyte Populations

**Figure 2:**
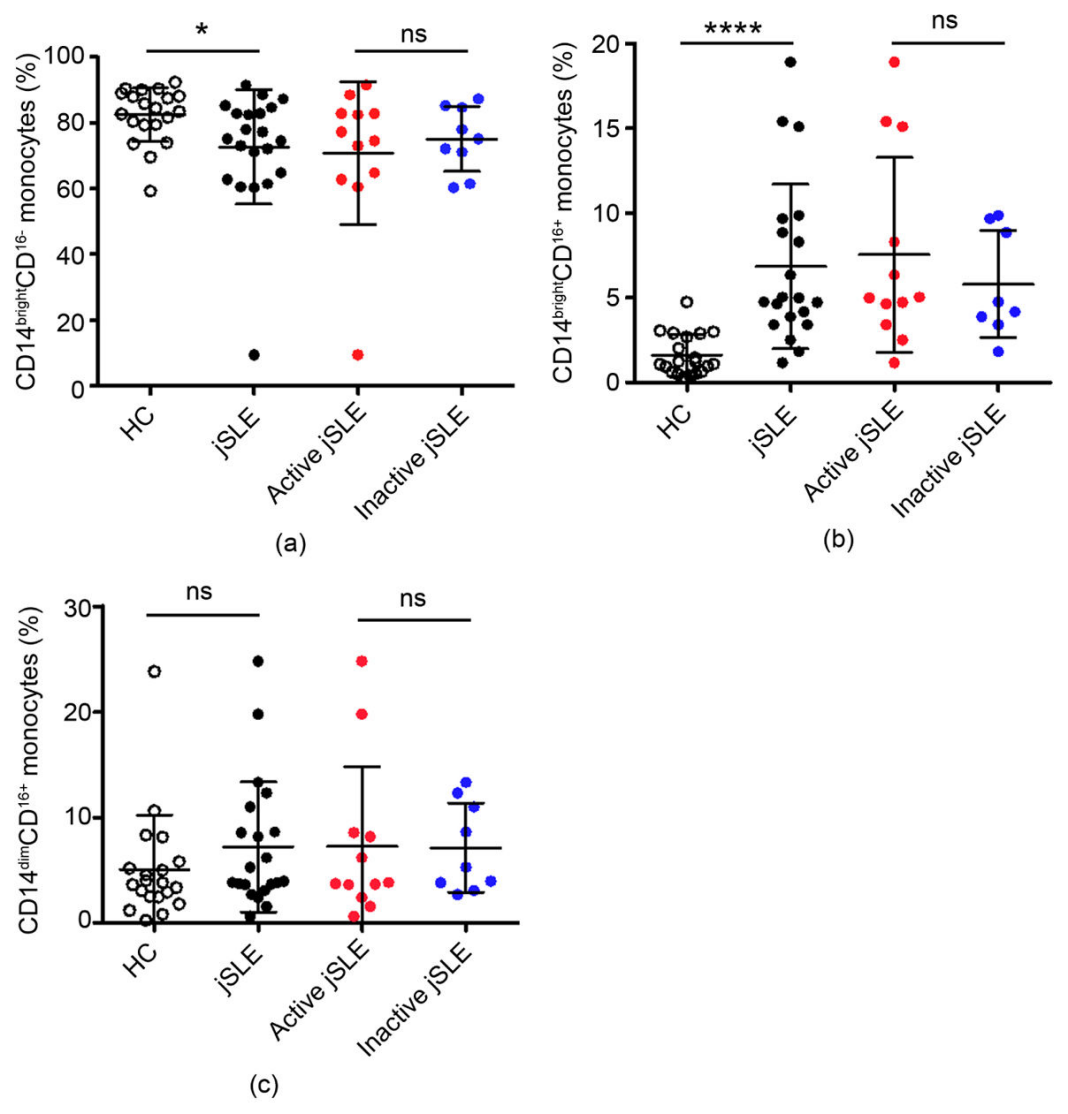
Frequencies of peripheral blood monocytes in juvenile-onset SLE patients and healthy controls (HC). (a)–(c) Comparisons of proportions (% of total CD14^+^ cells) of (a) CD14^bright^CD16^−^, (b) CD14^bright^CD16^+^, and (c) CD14^dim^CD16^+^ monocytes in HC to those in jSLE patients, and between active and inactive disease. Horizontal lines and error bars show median and interquartile range; *p < 0.05; ****p < 0.0001; (ns) not significant.

NKG2DL are present on normal monocytes presumably contributing to regulatory crosstalk with NKG2D^+^ lymphocytes [19] [20] [21] [22]. With no evidence for aberrant NKG2DL presence among lupus B cells we thus considered myelomonocytic cells as possible NKG2DL source for NKG2D^+^CD4 T cell activation and expanded the flow cytometry-based screen to lupus monocytes. All but one (jSLE044; excluded due to limiting cell numbers) patient and control PBMC samples were tested. Monocytes were classified as classical, intermediate, and non-classical subsets based on expression of CD14 and CD16 (Supplementary Figure S2; [23]). Proportions of classical (CD14^bright^CD16^−^) monocytes were decreased, and those of intermediate (CD14^bright^CD16^+^) increased in jSLE compared to control samples; non-classical (CD14^dim^CD16^+^) monocytes were similar in both groups (Figures 2(a)–(c); [24]). None of these changes correlated with disease activity scores (Figures 2(a)–(c)).

**Figure 3:**
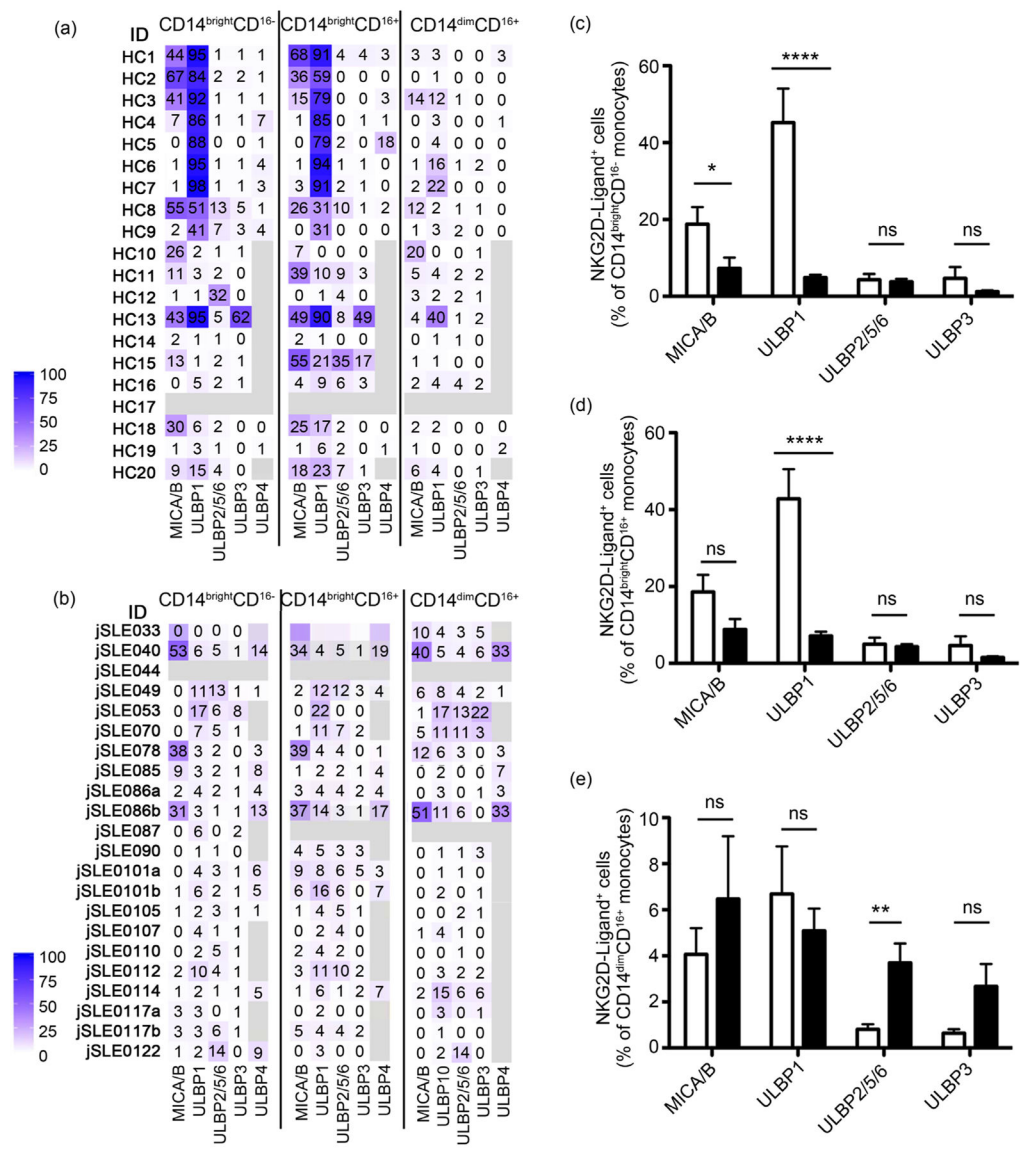
NKG2DL phenotypes of peripheral blood monocytes in juvenile-onset SLE patients and healthy controls (HC). (a) (b) Heat map display of proportions (numbers in individual squares) of CD14/CD16-defined monocytes expressing the indicated ligands in each HC (a) or jSLE patient (b) sample. Bars display color grading. Light grey indicates no data; (ID) identification. (c)–(e) Graphic display of proportions (in %) of (c) CD14^bright^CD16^−^, (d) CD14^bright^CD16^+^, and (e) CD14^dim^CD16^+^ monocytes expressing the indicated ligands. Open and black bars represent data from HC and patients, respectively. *p < 0.05; **p < 0.01; ****p < 0.0001; (ns) not significant.

As with jSLEB cells, NKG2DL profiles of patient monocytes resembled the normal expression pattern with substantial variability among individuals and at least one, and frequently two or more ligands expressed (Figure 3(a) and Figure 3(b)). Unlike with B cells, however, all patient monocyte populations differed from controls in the extent to which NKG2DL, and in particular MICA/B and ULBP1, were expressed (Figures 3(a)–(e)). jSLE monocyte populations contained, in part significantly, lower frequencies of ULBP1 positive cells than their corresponding controls. MICA/B positive cells were less frequent among the classical CD14^bright^CD16^−^ and intermediate CD14^bright^CD16^+^ monocyte subsets compared to controls. Cells expressing ULB2/5/6 or ULBP3 were generally rare but slightly more frequent among jSLE CD14^dim^CD16^+^ monocytes. Thus, unlike with B cells, jSLE monocytes displayed disease-associated NKG2DL phenotypes although none correlated with disease activity or kidney involvement (data not shown). Due to limiting sample sizes testing of ULBP4 expression was sporadic.

Lupus antigen presenting cells have functional impairments such as an inability to induce the immuneregulatory ligands PD-L1 and CD80 [25] [26]. The apparent lack of ULBP1 and/or MICA/B expression on most jSLE monocytes cells may reflect a similar defect. Hence, adherence-enriched jSLE and control monocytes were cultured in the presence of stimuli known to induce NKG2DL in normal myeloid cells and monitored for surface NKG2DL expression over time [19] [20] [21]. GM-CSF or LPS treatment resulted in induction of MICA/B and ULBP1 in normal controls but had no effect with jSLE monocytes (Supplementary Figure S3; [19] [20] [21]). ELISA of culture supernatants for soluble NKG2DL (sMICA, sMICB, sULBP1) was negative suggesting that the lack of surface NKG2DL on the jSLE monocytes was not due to enhanced ligand shedding [27]. Thus altogether, as with lupus B cells, lupus monocytes unlikely provide NKG2DL driving NKG2D^+^CD4 T cell proliferation. To ensure comprehensiveness, we also examined patient and control T cell and NK cell compartments for NKG2DL expression. Rare (<5%) NKG2DL-positive CD4 and CD8 T cells were detected in both cohorts with no prevalence for one or the other. NK cells (defined based on expression of CD56 and/or CD16) were NKG2DL-negative (data not shown).

### 3.3. Soluble MICB in Juvenile-Onset SLE Plasma and Urine Aligns with Frequencies of NKG2D^+^CD4 T Cells

**Figure 4:**
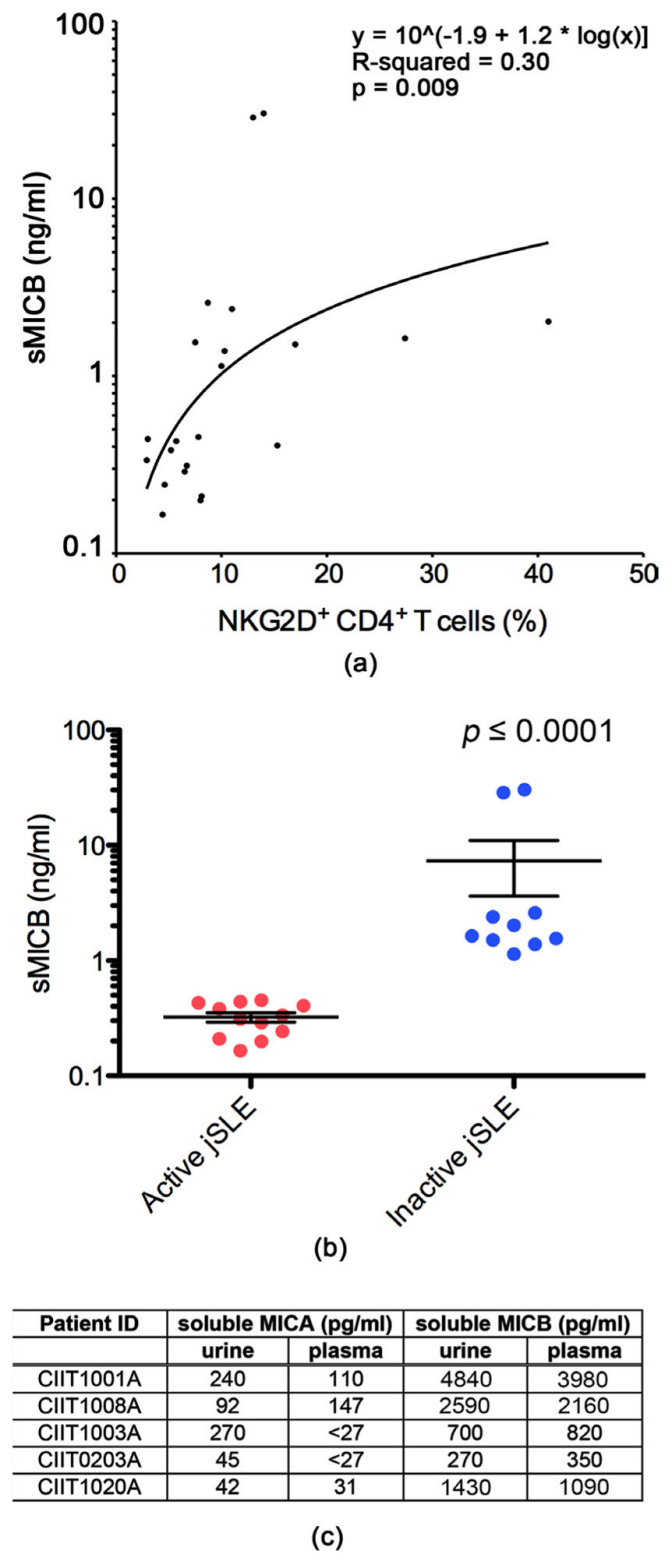
Presence of soluble MICB in plasma and urine from juvenile-onset SLE patients. (a) Overall data point distribution and statistical evaluation (y = 10 ^ [−1.9 + 1.2 * log(x)], R-squared = 0.3; p = 0.009) of soluble MICB plasma concentrations (ng/ml; log scale) in relationship to frequencies of NKG2D^+^CD4 T cells in the 22 jSLE patient samples studied. (b) Comparisons of soluble MICB plasma concentrations (ng/ml; log scale) in jSLE patients with active disease to those of patients with inactive disease. Horizontal lines and error bars show median and interquartile range. (c) Tabulation of soluble MICA and soluble MICB concentrations (pg/ml) in urine and paired plasma from five jSLE patients. Patient ID = jSLE patient identification.

Continuing the search for NKG2DL positive environments we resorted to screening for presence of soluble NKG2DL in matched jSLE patient plasma as surrogate readout for NKG2DL expression elsewhere [3] [12]. Although our earlier study found no significant association between frequencies of NKG2D^+^CD4 T cells and soluble MICA (sMICA) plasma concentrations we revisited this issue and extended the analysis to other soluble NKG2DL. Plasma samples matching all 22 patient PBMC specimens were screened by ELISA for sMICA, sMICB, and sULBP1. 14 of these samples were positive for sMICA yet concentrations generally low (picogram range) and unaligned with NKG2D^+^CD4 T cell frequencies (data not shown). sMICB was detectable in all 22 plasma samples at concentrations that varied widely (range: 199 pg/ml – 30.3 ng/ml) among samples and displayed a significant positive trend relationship with NKG2D^+^CD4 T cell proportions (Figure 4(a)). sMICB plasma values also correlated negatively with disease activity scores suggesting clinical relevance (Figure 4(b)). ELISA for sULBP1 was negative throughout. Although sMICB plasma values were independent of presence or absence of overt renal disease we considered kidney as the most likely site of aberrant NKG2DL expression and putative origin of NKG2DL shedding [28]. Because of lack of access to kidney biopsies we examined patient urine instead for the presence of soluble NKG2DL (sMICA, sMICB, and sULBP1). Urine specimens matching the 22 patient and 20 control PBMC and plasma were not available. We thus examined urine and paired plasma from an additional five jSLE patients (Table 1) and age-matched controls by sMICA and sMICB ELISA. All patient—but none of the HC—derived samples were positive for sMICB with concentrations in the two specimen types well aligned (Figure 4(c)). sMICA was detected in all jSLE urine samples and in three of the paired plasmas (Figure 4(c)). Extending these observations to adult lupus, paired urine and plasma specimens from three SLE patients also contained low concentrations of sMICA and abundant sMICB. ELISA for sULBP1 was negative throughout. Filtration of plasma sMICA/B in the kidney is unlikely as control urine samples from patients with MICA/B expressing tumors and abundant plasma sMICA/B were negative for sNKG2DL (data not shown). Altogether, these results point towards the kidney as the site of aberrant NKG2DL expression in jSLE with both cell surface and soluble ligands possibly driving the NKG2D^+^CD4 T cell population expansions typical for this disease.

## 4. Discussion

Aberrant NKG2DL presence is thought to be relevant in lupus disease regulation but the tissues and cell types involved have not been defined [3] [28] [29] [30]. This study’s goal was to identify the cellular contexts that might provide NKG2DL for stimulation of the immunosuppressive NKG2D^+^CD4 T cell subset that has been implicated in modulating jSLE disease activity [3]. Although earlier observations with NKG2D^+^CD4 T cells in healthy individuals pointed towards the B cell compartment as possible site of enhanced NKG2DL presence, there was no evidence for a disease-associated increase of NKG2DL-positivity among jSLE B cells. This was somewhat unexpected, as NKG2DL induction among lupus B cells would be consistent with NKG2D-mediated co-stimulation driving proliferative expansions of autoreactive and B cell antigen-specific NKG2D^+^CD4 T cell populations [3]. jSLE monocytes, largely devoid of NKG2DL, also emerged as unlikely source of NKG2DL-mediated NKG2D^+^CD4 T cell stimulation. However, patient plasma and matched urine samples were positive by ELISA for the soluble form of MICA and MICB, suggesting that kidney and/or peripheral blood might constitute the NKG2DL positive microenvironment driving NKG2D^+^ CD4 T cell population expansions [3] [12]. Positive trend relationships between sMICB plasma concentrations and proportions of NKG2D^+^CD4 T cells support this notion [12]. Moreover, sMICB values were inversely correlated with disease activity. As with an earlier study, sMICA plasma concentrations were independent of NKG2D^+^CD4 T cell frequencies possibly due to masking of ELISA by anti-MICA autoantibodies [3]. However, why such a mechanism would preferentially affect MICA over MICB remains unexplained. Whether or not the apparent quantitative prevalence of sMICB compared to sMICA reflects a true biological phenomenon or simply differential sensitivities of the respective ELISA remains equally unknown [28]. NKG2DL expression in, and shedding from jSLE kidney in the absence of overt nephritis is not surprising as even subclinical alterations of tissue homeostasis can lead to induction of NKG2DL expression [7].

The lack of and/or failure to induce MICA/B and/or ULBP1 in jSLE monocytes is consistent with current concepts of lupus-related myeloid cell abnormalities [24] [25] [26]. However, enhanced cell segregation to inflammatory sites may also contribute to the reduced ULBP1- and/or MICA/B-positivity cells among jSLE monocytes, as sequestration of myeloid cells to renal tissue has been described for patients with active lupus nephritis [23] [31]. However, we found no correlation between frequencies of NKG2DL-positive monocytes and presence or absence of renal disease. Arguing against immune-mediated depletion via auto-anti-antibody-mediated cytotoxicity, viability of peripheral blood cells from healthy donors was unaffected by incubation with active jSLE patient sera (data not shown). Medication effects on NKG2DL are unlikely as use of immunosuppressive drugs was comparable among all patients (Table 1; [25]).

In addition to the disease-related changes, this study uncovered a previously underappreciated inter-individual variability in NKG2DL expression by peripheral blood B cells and monocytes [19] [20].

## 5. Conclusion

In summary, although our study does offer insights into the distribution of NKG2DL in juvenile-onset lupus patients, it fails to directly pinpoint the precise tissue source for the soluble NKG2DL present in patient plasma and urine. An additional limitation is the relatively small number of matched plasma and urine samples studied. Analysis of a larger patient cohort including more extensive serial sampling is thus desirable as it may lead to the identification of soluble NKG2DL in urine as a disease activity biomarker.

## Supplementary Material



## Figures and Tables

**Table 1:** SLE patient characteristics.

Subject	Age at onset (yr)	Age at draw (yr)	Gender	SLEDAI	Kidney involvement	Prednisone^a^ (mg/kg/day)	Other immunomodulatory medications*
jSLE033	14	16.3	F	22	Yes	0.19	HCQ, Dipyridamole
jSLE040	7	13	F	18	Yes	0.36	HCG, MMF, Dapsone, CyC, Abatacept
jSLE044	17	19.6	F	0	No	0.08	HCQ, MMF
jSLE049	12	13.7	F	2	No	0.32	HCQ
jSLE053	11	13.9	F	4	No	None	HCQ
jSLE070	16	15.9	F	8	Yes	1.9	HCQ, MMF
jSLE078	12	17.2	F	0	Yes	None	HCQ, AZT, Dapsone
jSLE085	15	14.9	F	12	No	None	None
jSLE086a	11	12.7	F	6	No	0.73	HCQ, MMF
jSLE086b		13.7		0	No	None	HCQ, MMF
jSLE087	15	16.5	F	2	Yes	0.11	HCQ, AZT, Mesalamine
jSLE090	15	18.2	F	4	Yes	None	None
jSLE101a	16	16.5	F	22	Yes	1.23	HCQ
jSLE101b		17.6		8	Yes	0.1	HCQ, MMF
jSLE105	15	16.3	F	7	No	None	HCQ, MMF
jSLE107	14	17.4	F	4	No	None	None
jSLE110	11	11.9	F	29	Yes	1.56	HCQ, AZT
jSLE112	11	12.4	F	4	No	None	HCQ, AZT
jSLE114	13	16.9	M	25	Yes	0.25	HCQ, AZT
jSLE117a	14	14.8	F	10	Yes	0.24	HCQ, MMF, Dapsone
jSLE117b		15.1		0	Yes	0.17	HCQ, MMF, Dapson
jSLE122	15	15.2	F	9	No	None	None
CIIT1001A	13	15	F	2	No	0.5	HCQ, MMF
CIIT1008A	15	18	F	2	Yes	0.2	HCQ, MMF, Rituximab, Tacrolimus
CIIT1003A	13	19	F	7	Yes	0.13	HCQ, AZT
CIIT0203A	7	16	F	22	Yes	None	None
CIIT1020A	15	15.2	M	2^b^	No	0.5	HCQ, MMF

a Therapeutic regimens including prednisone > 2 mg/kg/day and/or mycophenolatemofetil (MMF), cyclophosphamide (Cyc), or azathioprine (AZT) were considered immunosuppressive.

HCQ = hydroxychloroquine.

b No SLEDAI available; clinically no disease activity, 2 reflects reduced complement levels.

## Non-Standard Abbreviations

NKG2DL: NKG2D ligand
MICA and MICB: MHC class I-related chains A and B
ULBP: UL-16 binding protein
SLE: systemic lupus erythematosus
jSLE: juvenile-onset SLE
HC: healthy control
