# Supplementary material for: Soluble MICB in Plasma and Urine Explains Population Expansions of NKG2D+CD4 T Cells Inpatients with Juvenile-Onset Systemic Lupus Erythematosus
Source: Open J Immunol. Author manuscript; Available in PMC 2018 Mar 1. (PMC5604888; doi:10.4236/oji.2017.71001)
Supplement: Supplementary file 1 [file NIHMS900221-supplement-supplement_1.pdf]

Appendix. Supplementary Data

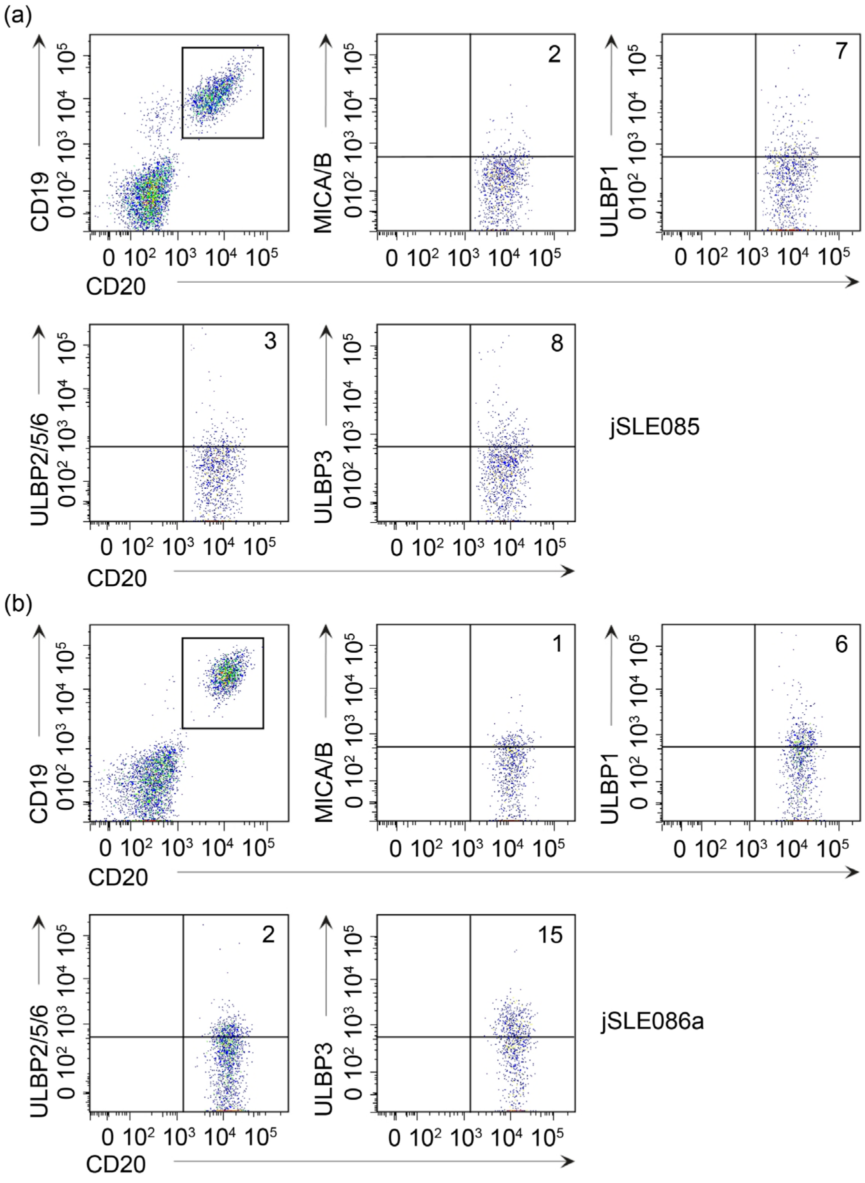

Figure S1. Example NKG2D ligand profiles of juvenile-onset SLE B cells. Flow cytometry dot plots displaying gating strategy (upper left dot plots) for, and anti-NKG2D ligand monoclonal antibody staining of CD19/CD20-defined peripheral blood B cells from juvenile-onset SLE patient jSLE085 (a) and jSLE086 (b). Numbers in quadrants represent % of total $CD19^{+}CD20^{+}$ cells.

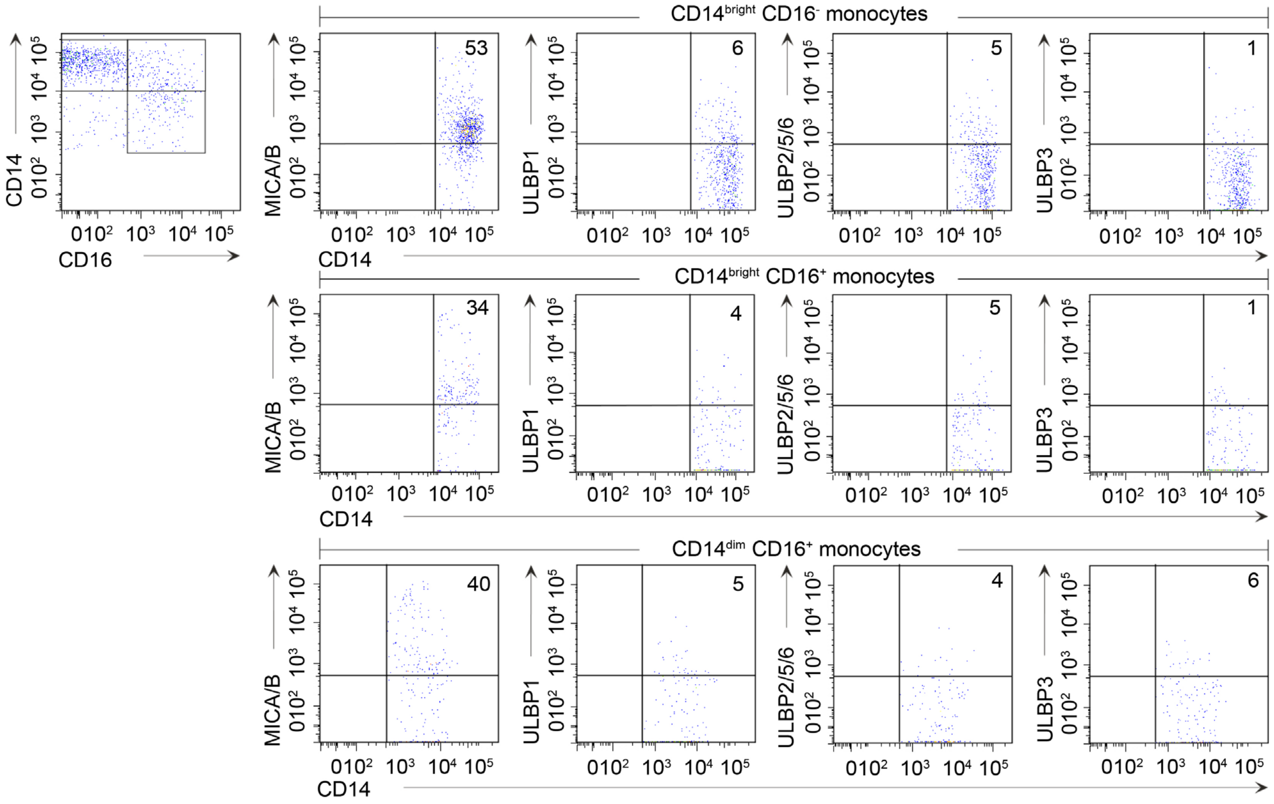

Figure S2. Example multicolor flow cytometry dot plots displaying gating strategy (upper left dot plot) for, and anti-NKG2D ligand monoclonal antibody staining of CD14/CD16-defined peripheral blood monocytes from a juvenile-onset SLE patient (jSLE040). Numbers in quadrants represent % of total $CD14^{bright}CD16^{-}$, $CD14^{bright}CD16^{+}$, and $CD14^{dim}CD16^{+}$ monocytes.

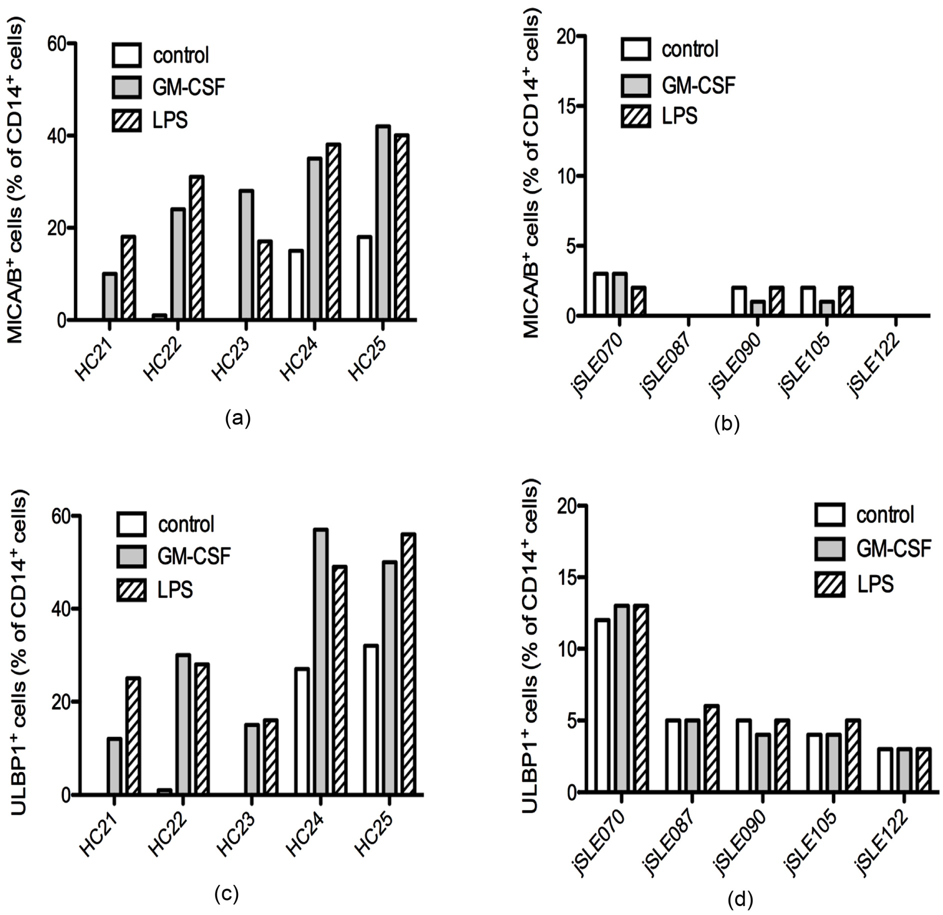

Figure S3. Failure to induce NKG2DL expression in juvenile-onset SLE monocytes. Graphic display of proportions (in %) of HC ((a) (c)) and jSLE ((b) (d)) $CD14^+$ monocytes expressing MICA/B ((a) (b)) and ULBP1 ((c) (d)) ligands after a 24 hr exposure to medium control (open bars), GM-CFS (grey bars), or LPS (shaded bars).

Scientific Research Publishing

Submit or recommend next manuscript to SCIRP and we will provide best service for you:

Accepting pre-submission inquiries through Email, Facebook, LinkedIn, Twitter, etc.
A wide selection of journals (inclusive of 9 subjects, more than 200 journals)
Providing 24-hour high-quality service
User-friendly online submission system
Fair and swift peer-review system
Efficient typesetting and proofreading procedure
Display of the result of downloads and visits, as well as the number of cited articles
Maximum dissemination of your research work

Submit your manuscript at: http://papersubmission.scirp.org/
Or contact oji@scirp.org
